# Injury intensifies T cell mediated graft-versus-host disease in a humanized model of traumatic brain injury

**DOI:** 10.1038/s41598-020-67723-x

**Published:** 2020-07-01

**Authors:** Miguel F. Diaz, Paulina D. Horton, Akshita Kumar, Megan Livingston, Amina Mohammadalipour, Hasen Xue, Max A. Skibber, Adesuwa Ewere, Naama E. Toledano Furman, Kevin R. Aroom, Songlin Zhang, Brijesh S. Gill, Charles S. Cox, Pamela L. Wenzel

**Affiliations:** 1 Children’s Regenerative Medicine Program, Department of Pediatric Surgery, McGovern Medical School, University of Texas Health Science Center at Houston, Houston, TX 77030 USA; 2 Center for Stem Cell and Regenerative Medicine, The Brown Foundation Institute of Molecular Medicine, University of Texas Health Science Center at Houston, Houston, TX 77030 USA; 3 Department of Integrative Biology and Pharmacology, McGovern Medical School, University of Texas Health Science Center at Houston, Houston, TX 77030 USA; 4 Department of Surgery, McGovern Medical School, University of Texas Health Science Center at Houston, Houston, TX 77030 USA; 5 Department of Pathology and Laboratory Medicine, McGovern Medical School, University of Texas Health Science Center at Houston, Houston, TX 77030 USA; 6 Present Address: School of Medicine, University of Texas Medical Branch, Galveston, TX USA

**Keywords:** Brain injuries, Inflammation, Mesenchymal stem cells, Graft-versus-host disease

## Abstract

The immune system plays critical roles in promoting tissue repair during recovery from neurotrauma but is also responsible for unchecked inflammation that causes neuronal cell death, systemic stress, and lethal immunodepression. Understanding the immune response to neurotrauma is an urgent priority, yet current models of traumatic brain injury (TBI) inadequately recapitulate the human immune response. Here, we report the first description of a humanized model of TBI and show that TBI places significant stress on the bone marrow. Hematopoietic cells of the marrow are regionally decimated, with evidence pointing to exacerbation of underlying graft-versus-host disease (GVHD) linked to presence of human T cells in the marrow. Despite complexities of the humanized mouse, marrow aplasia caused by TBI could be alleviated by cell therapy with human bone marrow mesenchymal stromal cells (MSCs). We conclude that MSCs could be used to ameliorate syndromes triggered by hypercytokinemia in settings of secondary inflammatory stimulus that upset marrow homeostasis such as TBI. More broadly, this study highlights the importance of understanding how underlying immune disorders including immunodepression, autoimmunity, and GVHD might be intensified by injury.

## Introduction

Traumatic brain injury (TBI) is a major contributor to long-term disability and mortality in children and adults, and, despite intensive efforts, there are no effective treatments for TBI^1,2^. Sequelae of TBI and soft tissue injuries resulting from polytrauma include changes in cerebral metabolism, excitotoxicity, induction of emergency hematopoiesis, infiltration of fluid and inflammatory cells into the brain, systemic cytokine storm and localized cytokine release, and activation of microglia, the resident macrophage of the central nervous system^3–8^. The immune system plays critical roles in promoting tissue repair and clearance of dying neurons during recovery from neurotrauma but is also responsible for the deleterious inflammation that kills healthy neurons. Systemic immunodepression and vulnerability to infection often follow the acute phase of TBI wherein hypercytokinemia and inflammation resolve, placing patients at increased risk of pneumonia and sepsis^9^. Thus, understanding the contribution of the immune system to recovery is critical to development of efficacious therapies for neurotrauma^10,11^.

Healthy brains normally have low numbers of surveilling peripheral immune cells within the parenchyma; nonetheless, peripheral immune cells do infiltrate the CNS, accumulate near areas of pathology, and can modify microglial polarization^12,13^. CD4^+^ and CD8^+^ T cells are recruited to the human brain after TBI and can be detected 3–26 days after injury^14,15^. Despite this increased accessibility to the central nervous system, CD4^+^ type 2 helper T (Th2) cells may not need to infiltrate the brain to modulate glial response, as they act via secretion of cytokines that circulate systemically^16–18^. Importantly, T cells capable of producing IFN-γ, like CD4^+^ type 1 helper T (Th1) cells, have been linked to increased microglial activation, including promotion of elevated microglial motility and phagocytic activity^19–21^. It was recently shown that programmed cell death-1 (PD-1) is upregulated by neurotrauma^22^. PD-1 targeting activates the epithelium of the choroid plexus to permit trafficking of monocytes into the brain, and this process depends upon IFN-gamma production by Th1 cells^23^. Not surprisingly, cytokines are altered in the brain parenchyma and peripheral blood in response to TBI, stroke, and neurodegerative disease^24,25^. Overexpression of cytokines in murine models has been shown to be neuroprotective in part through alternative activation of microglia, reducing neurodegeration in diseases such as Alzheimer’s, but the clinical consequences of cytokine perturbation in TBI are still incompletely understood^26–30^.

Bone marrow mesenchymal stromal cells (MSCs) possess unique potential as neurotrauma therapeutics in part because their innate function is to regulate the hematopoietic system during homeostasis and in response to injury and infection. In addition to supporting hematopoietic stem cell (HSC) self renewal in the bone marrow, MSCs are thought to actively home to damaged tissue, attract immune cells by release of chemokines, and repress T, B, NK, macrophage, and dendritic cell inflammatory phenotypes via direct contact and paracrine signaling. We and others have shown that learning and memory are improved by administration of MSCs after TBI. Specifically, our studies show that MSCs promote hippocampal neurogenesis and improved neurocognitive function in mice after TBI^31^. Consistent with these data, a meta-analysis of 28 other studies indicate that MSCs exert a positive effect on locomotor recovery^32^. While MSCs and MSC potency-enhancing factors improve outcomes in animal models associated with inflammation such as TBI, full restoration of cognitive function in animal models of TBI remains elusive. Our recent reports suggest that mechanical cues may contribute uniquely to the intracellular signaling that initiates MSC repression of inflammatory phenotypes^33,34^. Exposure of human MSCs to fluid shear stress typical of frictional forces present on the luminal lining of arteries (15 dyne/cm^2^) stimulates a broad range of anti-inflammatory signaling (via *COX2*, *TSG6*, *IL1RN*, and *HMOX1)* and enhances MSC potency in suppression of cytotoxic TNF-α production by activated immune cells from the spleen^35^. In particular, we find evidence for prostaglandin E_2_, a metabolic product of COX2 enzyme activity, as a key mediator of shear-amplified efficacy and improved therapeutic potency^33,36^.

We utilize a humanized mouse model of TBI to examine chief components of the human immune system likely to contribute to outcome following trauma. Unexpectedly, we find that graft-versus-host interactions in the bone marrow and variation in human chimerism between animals complicates interpretation of immune response to neurotrauma. Despite these limitations, the model suggests that TBI exacerbates alloreactivity and rejection of host marrow and/or host niche components, leading to marrow destruction. The effect was more pronounced in injured mice that did not receive MSC therapy, suggesting that physiologic stress associated with injury could exacerbate pathology but that MSCs conferred some protection from TBI-induced immune activation in the marrow. Herein, our data demonstrate a role for T cells in bone marrow fitness following neurotrauma and suggest that, with judicious use, the humanized mouse could enable identification of human immune subsets important for neural protection and repair, as well as those that contribute to systemic disease and increased susceptibility to infections that cause patient morbidity after TBI.

## Methods

### Transplantation of human hematopoietic cells

Newborn NOD-*scid IL2Rɣ*^*null*^ (NSG) mice (Jackson Laboratory, Bar Harbor, ME) within 48 h of birth were exposed to sublethal irradiation (100 cGy). Three hours after myeloablative conditioning, mice were anesthetized on ice and were infused via facial vein with a total of 2.5 × 10^5^ primary human umbilical cord blood CD34^+^ cells (Stemcell Technologies, Cambridge, MA). Briefly, commercially enriched CD34^+^ cells were thawed from cryopreservation and resuspended in 15 µl of sterile saline per neonate for intravenous transplantation using a Hamilton glass syringe, as reported in our prior study^37^. After cell infusion, pups were gently warmed and returned to the mother. All transplantation experiments were approved by and conducted in compliance with guidelines from the the Institutional Animal Care and Use Committee (IACUC) at the University of Texas Health Science Center.

### Bone marrow MSC derivation and culture

Bone marrow stromal cells were derived from whole bone marrow from independent human donors (AllCells, Alameda, CA). Mononuclear cells from whole bone marrow were enriched in the buffy layer of Ficoll-Paque. Cells were resuspended for immediate expansion in complete culture medium consisting of MEM-α (Thermo Scientific, Waltham, MA), 20% fetal bovine serum (Atlanta Biologicals, Flowery Branch, GA), 2 mM L-glutamine (Gibco, Waltham, MA), 100 units/ml penicillin (Gibco, Waltham, MA), and 100 μg/ml streptomycin (Gibco, Waltham, MA). Nonadherent cells were removed after 2 days. Adherent colonies were expanded further and frozen as Passage 1. MSCs were profiled for expression of surface markers consistent with minimal guidelines established by the International Society for Stem Cell Therapy^38^, as reported previously^33^. Thawed MSCs were plated at 1 × 10^5^ cells/ml, and medium was changed every 3 days. At 80% confluence, cells were passaged by treatment with TrypLE Express (Gibco, Waltham, MA) into IBIDI channels (μ-Slide I 0.4) at a density of 2–6 × 10^4^ cells/cm^2^ for mouse TBI experiments.

### Application of fluid wall shear stress (WSS)

Human MSCs were allowed to attach for 18 h on gas-permeable polymer coverslips within microfluidic channel slides (μ-slide I 0.4, IBIDI LLC, Fitchburg, WI). We applied unidirectional flow rates of 11.4 ml/min, corresponding to 15 dyne/cm^2^ laminar WSS on the culture surface, by peristaltic pump (Masterflex, Vernon Hills, IL) for 3 hr^35^. Flow rate required to achieve this force assumed steady laminar flow through a three-dimensional rectangular pipe ^39^. We determined WSS at the bottom center of the channel as ${\tau }_{w}=-\mu {\left.\frac{du}{dy}\right|}_{0}$, where $u$ is the linear velocity of fluid flow, $y$ is the position within the channel a distance of zero from the culture surface, and $\mu$ is the fluid dynamic viscosity. The value for $\mu$ was estimated as the viscosity of water at 20 °C (1.0 mPa sec), which closely approximates that of media at 37 °C. The average linear velocity was determined as $u= \frac{Q}{hw},$ where $Q$ is the volumetric flow rate (ml per min), $h$ is the height of the channel, and $w$ is the width of the channel. Static controls were plated in microfluidic slides under no flow conditions, with the exception of fluid displacement associated with manual medium change.

### Controlled cortical impact (CCI) and administration of MSC therapy

Experimental traumatic brain injuries were delivered on exposed brain in 5–8 month old male and female NSG mice by a Leica Impactor 1 CCI device. Injury was produced by administration of a single impact on the right parietal association cortex with a 3 mm impactor tip. Depth of deformation was 1.0 mm, and velocity was 5 m/s with 200 ms dwell time. Control (sham) mice were treated with cranial incision and craniotomy alongside animals undergoing CCI. MSC therapy included delivery of low passage (P2–5) bone marrow MSCs that were cultured under static conditions or preconditioned for 3 h with WSS of 15 dyne/cm^2^. MSCs (1 × 10^7^ cells/kg) suspended in culture medium were injected via tail vein within 2 h of WSS exposure. Between 4 and 8 experiments conducted across different days were used to generate data for analysis of immune cells in the brain and hematopoietic tissues. Between 6 and 15 mice were prepared per treatment group. Each mouse appears as a single data point in graphical displays. All animal experiments were approved by and conducted in compliance with guidelines from the IACUC at the University of Texas Health Science Center.

### Isolation of tissues

Peripheral blood and bone marrow isolation was performed as previously described^40^. Briefly, blood was collected from the retroorbital plexus at 6-week intervals after human umbilical cord CD34^+^ cell transplantation. White blood cells were enriched by 1% dextran sulfate-PBS-EDTA separation and treatment with RBC lysing buffer (Sigma-Aldrich, St. Louis, MO). Long bones of both legs were isolated and cleaned of all muscle 7 days after surgical procedures. One pair of femur and tibia were placed in 4% paraformaldehyde for histopathology and the other pair were crushed in PBS with a mortar and pestle, incubated with RBC lysing buffer, followed by filtration through a 70 μm cell strainer in preparation for flow cytometry. Lymph nodes were digested with a mixture of collagenase and DNAse and filtered through a 70 μm cell strainer. Spleens were macerated in 2% FBS-PBS for filtration in a 70 μm cell strainer, and leukocytes were enriched by removal of erythrocytes with RBC lysing buffer. The brain was collected 7 days after injury, and microglia were isolated using a Neural Dissociation kit (Miltenyi Biotec, Waltham, MA). The resulting single cell suspension was washed and cleaned of myelin using 30% Percoll in HBSS, followed by a CD11b/c enrichment using a MACS kit (Miltenyi Biotec, Waltham, MA). The myeloid-enriched cells were then prepared for flow cytometric analysis using the protocol described below.

### Flow cytometry

Subsets of human and murine cells were identified by immunostaining for flow cytometric analysis immediately following isolation of tissues. Cell preparations for analysis of T regulatory cells were fixed and permeabilized, whereas, all other panels were unfixed samples. Antibodies for detection of chimerism, T regulatory cells, murine myeloid derived suppressor cells (MDSCs), human MDSCs, and microglia are outlined in Table S1. Unfixed cells were resuspended in 2% FBS-PBS buffer containing DAPI (1 µg/ml) and fixed cells were placed in wash buffer prior to analysis on a 3-laser Becton Dickinson LSR II flow cytometer. Gating for all panels was determined with fluorescence minus one controls.

### Statistical analyses

All data were analyzed with SigmaPlot 12.5 for statistical significance and are reported as individual points for each independent biological sample where feasible, with mean ± SEM. All data were evaluated for normality and variance to determine appropriateness of parametric versus non-parametric statistical tests. One-way ANOVA and either the Holm-Sidak method or Tukey test for multiple comparisons were used to evaluate differences between cell numbers, frequencies of immune cell subsets, and spleen size. Linear regression was used to evaluate the correlation between immune cell frequencies and spleen size. Pearson’s chi-square was used to assess whether bone marrow loss was independent of chimerism. The t-test or Mann–Whitney Rank Sum test was used to evaluate the relationship between marrow aplasia and chimerism. Significance levels of p < 0.05, < 0.01, or < 0.001 are denoted in graphs by a single, double, or triple asterisk, respectively.

## Results

**Figure 1:**
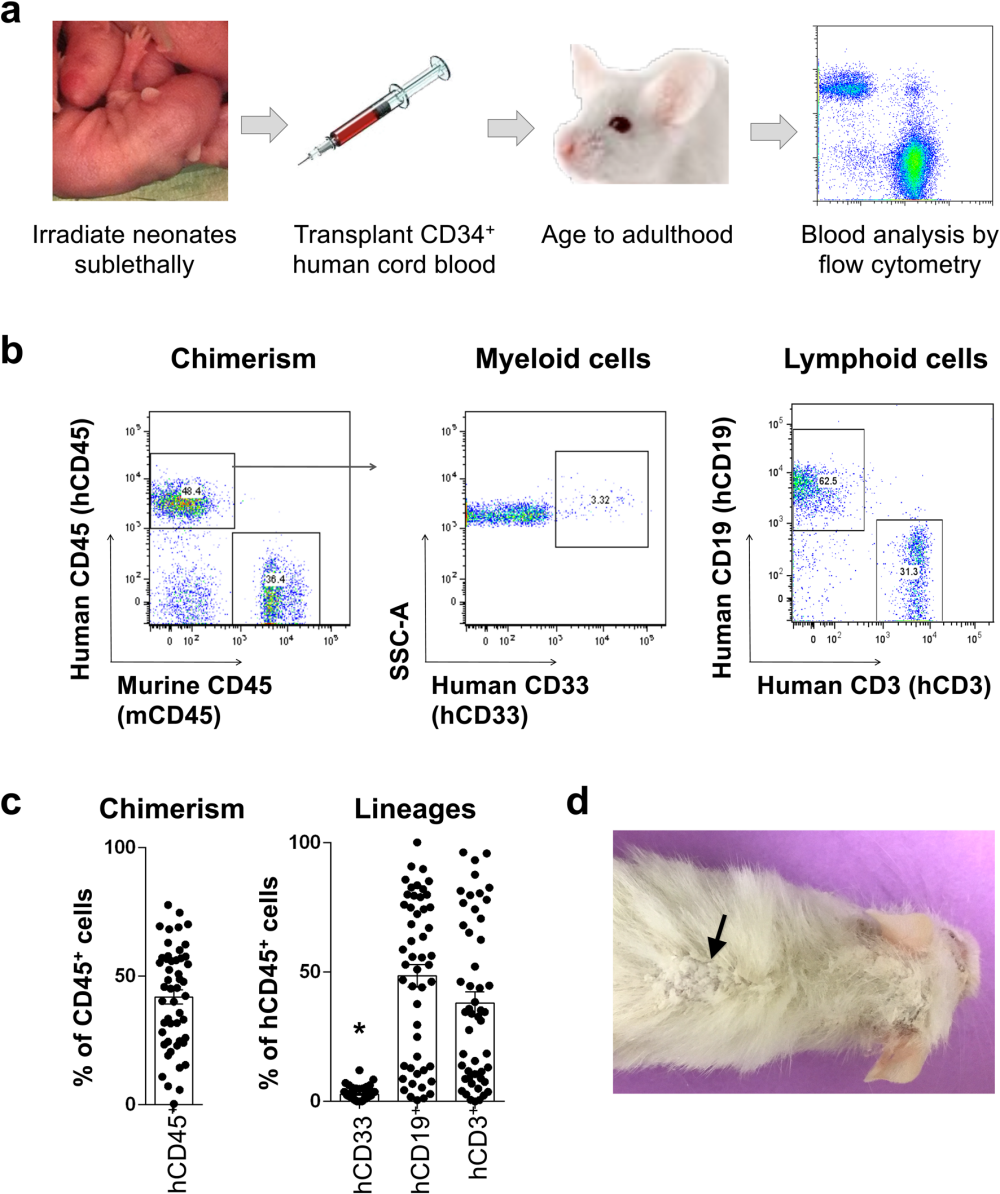
Human T and B cells predominate in grafts from human cord blood. **(a)** Immunocompromised NSG mice were transplanted with CD34^+^-enriched human cord blood as neonates via facial vein and monitored for human chimerism in the peripheral blood. **(b, c)** Human and mouse leukocytes were distinguished in the peripheral blood by surface expression of human CD45 and murine CD45. Blood lineages were detected with human-specific antibodies to CD33 (myeloid), CD19 (B cells), and CD3 (T cells). Analysis revealed robust contribution of CD34^+^ cells to T and B cells but poor reconstitution of myeloid cells in the peripheral blood (Kruskal–Wallis one-way ANOVA with Tukey test, *p < 0.05). Data points represent 50 individual mice at 2–5 months of age, and error bars indicate standard error of the mean (SEM). **(d)** Photograph of a humanized mouse reveals large patches of white, flaking skin apparent on the back (arrow) and head.

To understand the human immune response to neurotrauma, we first engrafted immunocompromised mice following sublethal irradiation with human HSCs to reconstitute a human immune system in the mouse. The NSG mouse model bears compound mutations that cause deficiency of mature T cells, B cells, and natural killer cells as well as defects in macrophages, dendritic cells, complement, and cytokine signaling. These insufficiencies make NSG tolerant of primary human cells, minimize competition from murine immune cells, and permit robust long-term multilineage human HSC engraftment^41^. T cell development depends upon epithelially derived interleukin 7 (IL-7) and can be supported by coadministration of IL-7 or, alternatively, engraftment into the neonate^42,43^. Thus, NSG were transplanted as neonates with CD34^+^-enriched human cord blood HSCs and monitored for chimerism into adulthood (Fig. 1a). Peripheral blood was collected at 6-week intervals and analyzed for surface expression of murine and human CD45 to measure chimerism of the immune system. Human T cells, B cells, and myeloid cells were identified by human-specific antibodies to CD3, CD19, and CD33, respectively (Fig. 1b). Human stem cells predominantly reconstituted T and B lymphocytes, with low-level contribution to myeloid cells (Fig. 1c). Mice with chimerism of less than 12.5% in the blood were culled and removed from the study. Sixteen of fifty mice died or were culled between 5.5 and 7 months prior to any surgeries. These animals, as well as several others, showed signs of clinically significant graft-versus-host disease (GVHD) including weight loss, reduced activity, postural changes such as hunching, fur ruffling, and skin that was noticeably white, wrinkled, and flaking, consistent with chronic GVHD of the skin in humans^44^ and reports of GVHD in NSG mice^45^ (Fig. 1d). GVHD has been shown previously in humanization of the NSG mouse and is most pronounced in animals provided human cytokines and/or microenvironments supportive of human blood cell development^46,47^. Based upon these data and reports in the literature, we postulated that this humanized mouse model could be leveraged to examine human adaptive immune cells but might exhibit symptoms of GVHD that could make it challenging to draw meaningful conclusions about how human cells modulate inflammation after TBI.

**Figure 2:**
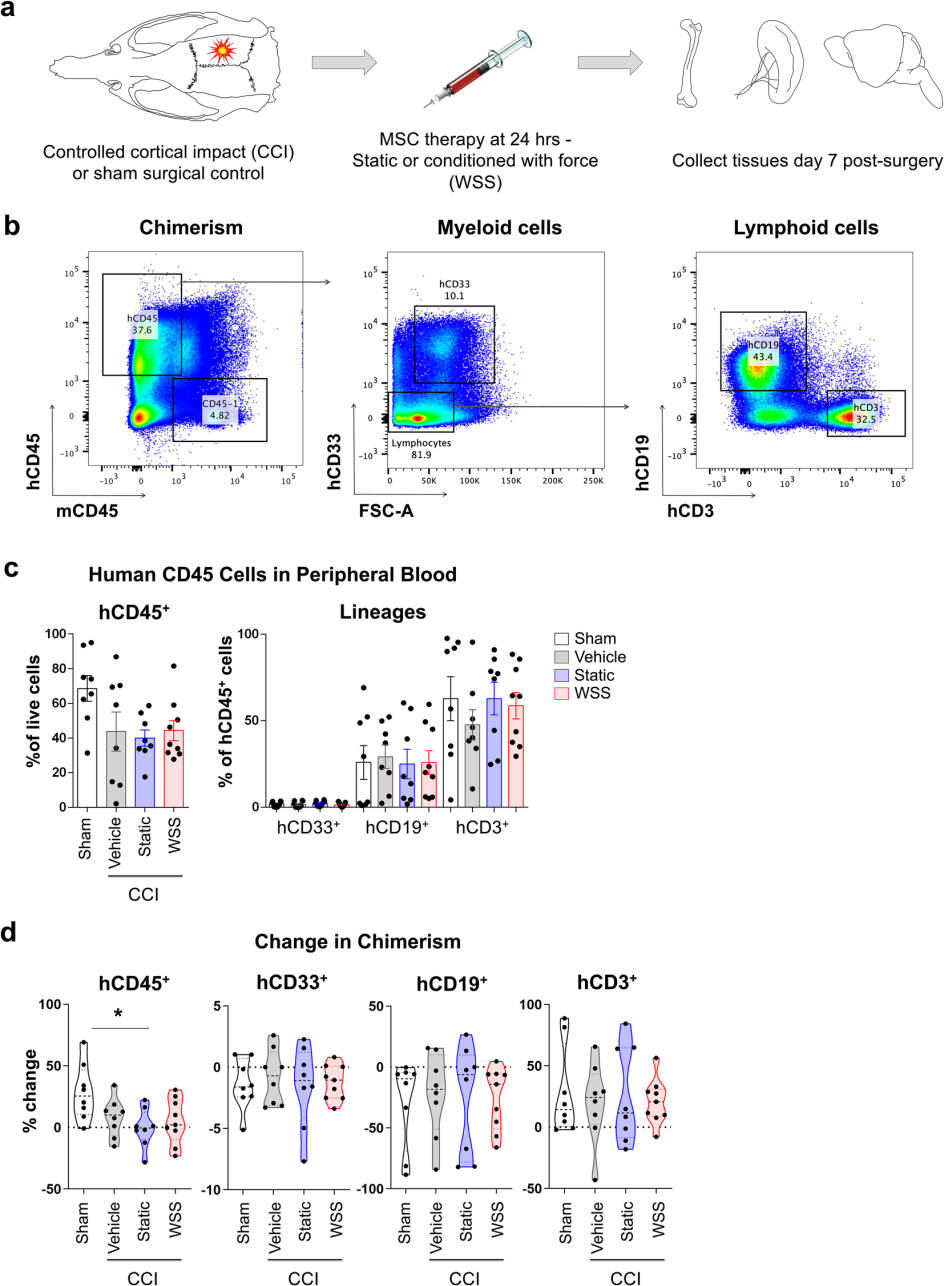
Human chimerism is reduced after CCI. **(a)** Traumatic brain injury (TBI) was administered by CCI to the right parietal association cortex between bregma and lambda. Mice received intravenous vehicle or MSCs cultured under static conditions or preconditioned transiently with 3 h exposure to WSS. Tissues were collected 7 days after injury. **(b, c)** Peripheral blood was analyzed for surface expression of mCD45 and hCD45 7 days after injury, along with human lineage markers. **(d)** Frequency of hCD45^+^ cells is decreased in the periphery after injury (One-way ANOVA with Holm-Sidak test, *p = 0.02). Pre-injury chimerism from 1–3 months before injury was used to calculate percent change. Lymphocyte production shifted with age from hCD19^+^ B cells to greater numbers of CD3^+^ T cells in all treatment groups during the course of 1–3 months. Data from 8–9 individual mice per group are shown, and SEM is indicated by error bars.

Following maturation to 6–8 months, humanized mice were randomized for TBI by CCI or sham surgical control that included craniotomy and all procedures except CCI (Fig. 2a). Injured mice received therapeutic doses of MSCs (1 × 10^7^ cells/kg) or vehicle control (media only) 24 h after surgery. MSCs were either cultured under standard (static) conditions or preconditioned by fluid WSS for 3 h prior to recovery for intravenous infusion. Animals receiving one of these four treatments (sham and three CCI groups: vehicle, static-cultured MSCs, WSS-preconditioned MSCs) were euthanized at a sub-acute (7-day) post-procedure time point. One vehicle control mouse died at 6 days after injury and was found to have a partially calcified fracture to the left tibia. Similarly, a static cultured MSC mouse died 4 days after injury. Non-humanized C57BL/6 mice underwent CCI or sham surgery for evaluation of a subset of outcomes. Chimerism and lineage composition of the peripheral blood of humanized mice was evaluated (Fig. 2b). The frequency of hCD45^+^ cells appeared elevated in sham animals, based upon comparison of each individual’s terminal peripheral blood chimerism relative to his or her baseline chimerism determined from 1–3 months prior (Fig. 2c). Whereas human chimerism had increased in non-injured sham controls, chimerism was blunted in injured individuals relative to the most recent pre-injury assessment, suggesting that TBI contributed to an overall decrease in human leukocyte numbers (Fig. 2d). Thus, the overall trend after injury is a slight reduction in human chimerism in all groups that underwent CCI. That the static MSC group was the only CCI treatment that was significantly different is most likely the result of the randomized assignment of mice to groups, regardless of their pre-injury variability in chimerism. In other words, mice assigned to the static MSC group had smaller variance in chimerism among individuals than the two other injury groups, thus contributing to a greater statistical power, which could have been due to initial segregation of the animals rather than a biological effect of the MSCs. Also observed was a decrease in B lymphocyte biased reconstitution across all groups (Fig. 2d). Instead, T cells were found to constitute the majority of the human graft at the terminal time point (Fig. 2c, d). This decrease in B cells over time is consistent with kinetics of engraftment reported previously^46^. MSC therapy did not appear to significantly alter chimerism of the animals.

**Figure 3:**
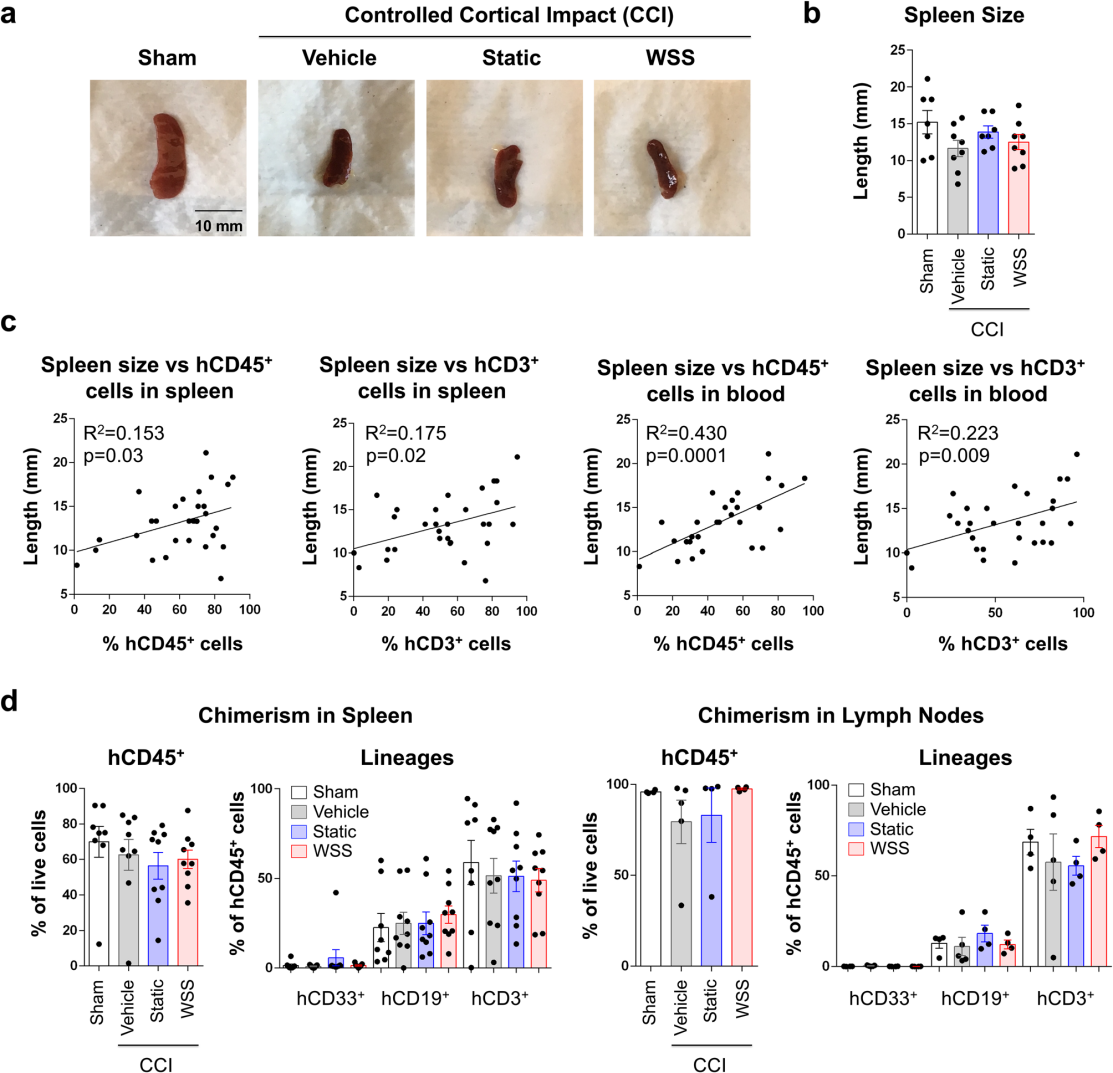
Spleen size correlates with human T cell chimerism. **(a)** Gross examination of spleens was conducted 7 days after surgery. **(b)** Measurement of spleen length of 7–8 mice per group reveals no significant reduction in size after injury (30 mice total). **(c)** Instead, spleen length was associated with frequency of hCD45^+^ and hCD3^+^ cells in the spleen and peripheral blood (Regression analysis, p < 0.05 for all comparisons). **(d)** No relationship exists between treatment group and human chimerism in the spleen or inguinal lymph nodes (8–9 mice per group for analysis of spleen and 4–5 mice per group for lymph nodes).

The spleen is a focal point for the immune response to brain injury, and spleen size decreases rapidly after stroke and neurotrauma as immune cells enter into the blood circulation, migrate to the brain, and release pro-inflammatory cytokines into the CNS^48,49^. Lymphocytes, neutrophils, and monocytes traffic away from the spleen, resulting in splenic contraction 24 to 48 h after injury wherein they infiltrate into the brain via a compromised blood–brain-barrier. Spleen size is typically restored by 96 h, but NK cells, T cells, and monocytes persist in the brain^49^. Thus, we examined spleens from injured mice and found no statistical difference in size relative to sham controls, consistent with nearly complete recovery from injury by day 7 (Fig. 3a, b). In contrast, hCD45^+^ and hCD3^+^ T cell contribution to the spleen and peripheral blood positively correlated with spleen length (Fig. 3c; Supplementary Fig. S1; Pearson’s chi-square test, p < 0.05). No apparent relationship was found between treatment group and frequency of human cells in the spleen or lymph nodes (Fig. 3d). These data indicate that spleen size is predominantly determined by the overall level of human engraftment and could introduce variability into post-injury changes in spleen size.

**Figure 4:**
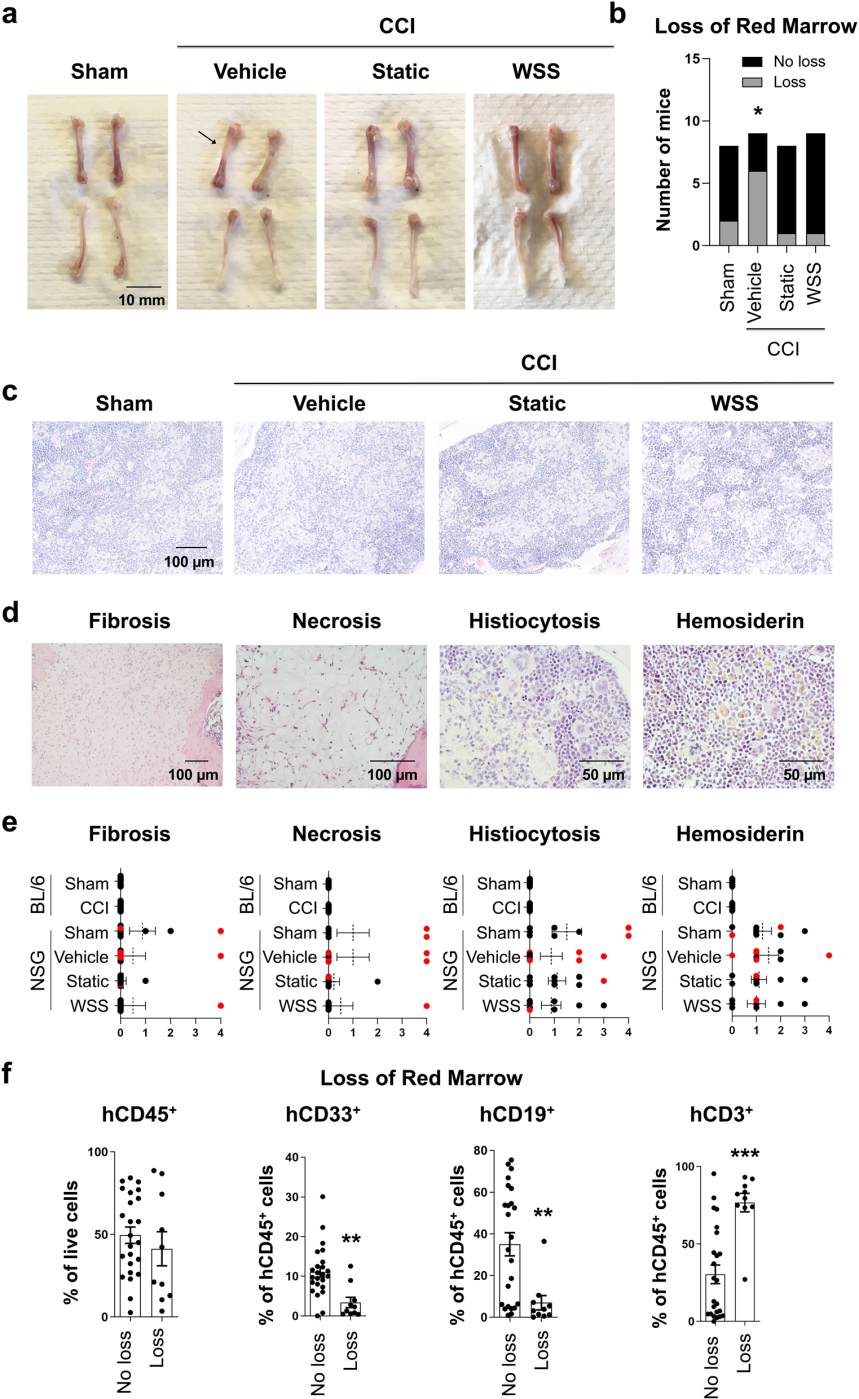
Brain injury is associated with bone marrow aplasia. **(a)** Evidence for loss of red marrow was apparent in several femurs collected from humanized mice 7 days after injury. Arrow indicates clearance of marrow in femur from vehicle control. **(b)** Graph represents quantification of mice with signs of bone marrow clearance from 8–9 mice per group (Chi-square test, *p = 0.03). **(c)** Histopathological examination revealed diffuse aggregates of histiocytes within the marrow of several animals, visible as eosinophilic areas among densely packed nucleated cells. Representative photomicrographs of femurs from each treatment group are shown. **(d)** Examples of necrosis, histiocytosis, and hemosiderin (brown iron deposits) are displayed. **(e)** Histopathological scores were based upon an ordinal numeric scoring system wherein 0 represented no abnormality and 4 displayed severe signs of pathology. NSG groups included 8–9 mice per treatment, and C57BL/6 mice included 9 mice per group. Red points represent mice scored as having loss of red marrow by gross observation. **(f)** Frequency of hCD3^+^ T cells in the bone marrow correlated with red marrow destruction (Mann–Whitney rank sum test, ***p < 0.001). Frequencies of hCD33^+^ myeloid or hCD19^+^ B cells were low in the bone marrow of mice with red marrow loss in one or both femurs as determined by gross observation (Mann–Whitney rank sum test, **p < 0.003). A total of 34 mice were included in these analyses and are plotted as individual points, along with mean and SEM.

Gross examination of the long bones of the skeleton revealed large white regions of bone resulting from absence of red marrow. Visible white sections of the diaphysis were particulary evident in the femurs, which appear red along the length of the bone in healthy animals (Fig. 4a). Injured mice that did not receive MSC therapy accounted for the majority of the observed cases (Fig. 4b). However, given that two sham animals were affected, this raised the possibility that marrow aplasia could be a result of host rejection by the human graft (or GVHD). Markedly reduced bone marrow cellularity was further confirmed by histopathological evaluation of the bones. Analysis revealed clusters of histiocytes in the marrow of mice from all groups, along with evidence of accumulation of hemosiderin, an iron complex deposit that can be caused by excessive erythrocyte destruction (Fig. 4c, d; Supplementary Fig. S2). Hemosiderin-containing hemophagocytic macrophages appeared brown by H&E staining. High histopathogical scores were evident in those individuals grossly determined to exhibit red marrow loss, indicated in red (Fig. 4e). Specifically, these individuals exhibited profound fibrosis and/or adipocytic infiltration of the marrow space, as well as necrosis and elevated histiocytosis. By contrast, these pathologies were not found in bone marrow from non-humanized C57BL/6 mice in either sham or CCI groups (Fig. 4e; Supplementary Fig. S2), further pointing to GVHD as a contributing etiology of the observed bone marrow destruction in NSG mice. Consistent with trends seen in the peripheral blood and spleen, the frequency of hCD45^+^ cells in the bone marrow trended toward reduction after injury but was not significantly different (Supplementary Fig. S2). Mice with exceptionally high levels of human T cell reconstitution more frequently presented with marrow loss; whereas, CD33^+^ and CD19^+^ frequencies were low (Fig. 4f). Recently, bone marrow aplasia related with acute GVHD in mice was reported to be alleviated by treatment with adipose-derived MSCs, pointing to a potential prophylactic application to ameliorate marrow suppression and infectivity after allogeneic HSC transplantation^50^. Not surprisingly, our data corroborates this finding, as recipients of MSC therapy experienced less severe marrow loss. Collectively, these data suggest that human T cell engraftment contributes to destruction of marrow, TBI exacerbates symptoms of GVHD in the bone marrow, and MSCs can partially suppress bone marrow aplasia associated with GVHD.

**Figure 5:**
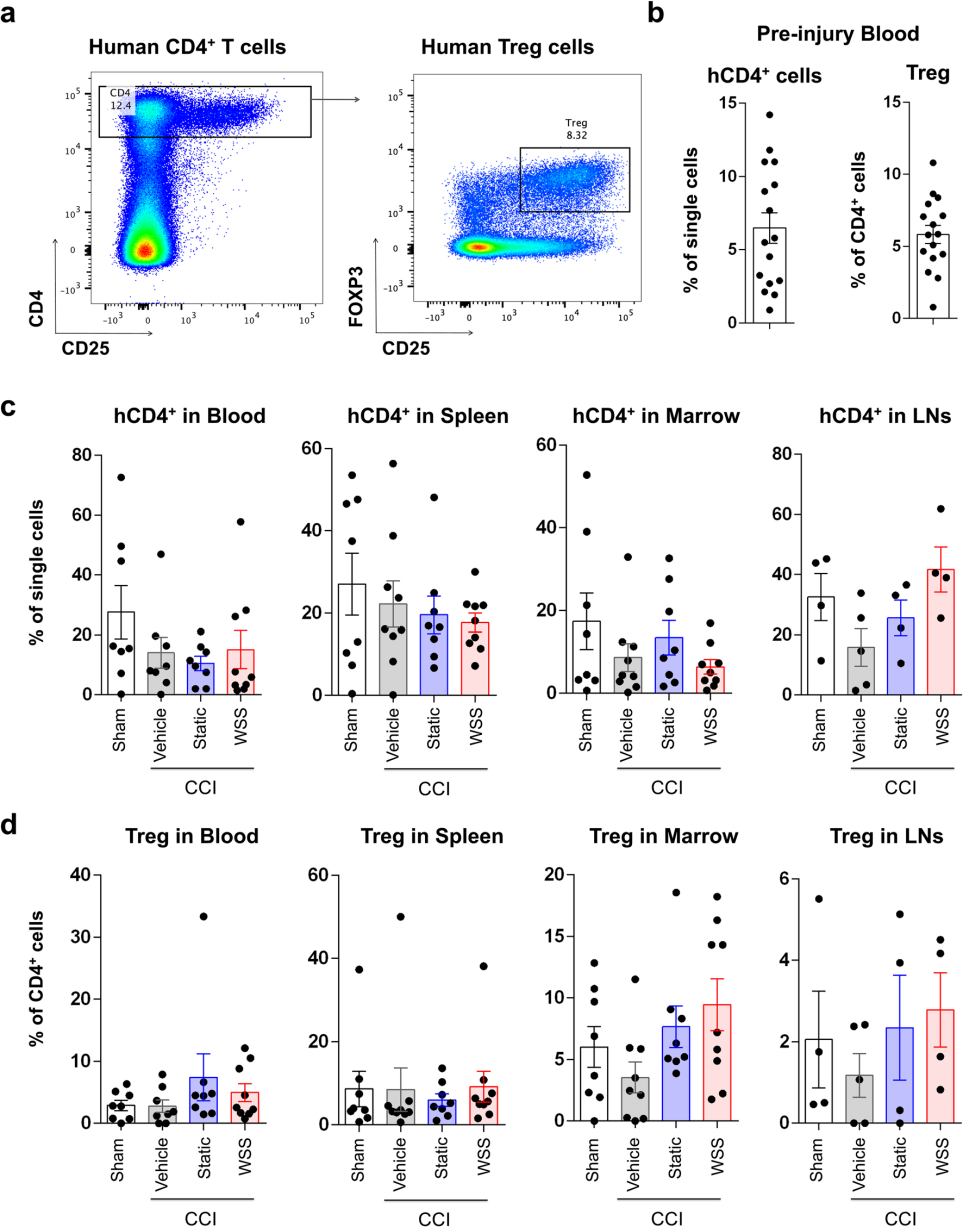
Cell therapy with MSCs elevates regulatory T cell frequencies in the bone marrow and lymph nodes. **(a)** Human CD4^+^ and regulatory T cells were measured before and 7 days after injury. Regulatory T cells were identified by CD4, CD25, and intracellular expression of FOXP3. **(b)** Frequency of hCD4^+^ T cells and Treg were quantified in the peripheral blood of 16 mice before assignment to a treatment group. **(c)** After injury, hCD4^+^ T cell frequency was generally lower but increased in the lymph nodes by therapy with MSCs preconditioned by WSS. 8–9 mice were analyzed per group. **(d)** Tregs were reduced in multiple hematopoietic organs after neurotrauma but increased after therapy with MSCs (n = 8–9 mice per group).

A prior report shows that CD4^+^ T cells in humanized mice can exhibit regulatory capacity^51^ and raises the possibility that stimulation of Treg by MSC therapy could alter bone marrow destruction after injury. We therefore examined T lymphocyte frequencies in various organs. CD3^+^ T and CD19^+^ B lymphocytes comprised approximately 38 ± 31% and 49 ± 31%, respectively, of human CD45^+^ cells in the peripheral blood before injury. Human CD4^+^ T cells accounted for 6.5 ± 4.1% of all single cells and, of hCD4^+^ cells, 5.8 ± 2.5% were regulatory T cells (Treg) (Fig. 5a, b). One to three months after the pre-injury measurement, the frequency of hCD4^+^ T cells was not significantly altered in the peripheral blood and spleen, although there was a non-significant trend (ANOVA, p < 0.1) toward decreased hCD4^+^ and Treg cells in the bone marrow and lymph nodes of injured vehicle control animals (Fig. 5c, d). Therapy with MSCs, including both static and force-conditioned MSCs, trended toward elevated Treg frequencies, although high variability prevented clear conclusions about the relationship between cell therapy and T cell decline and/or phenotype after injury. We predict from these data that CD4^+^ effector T cells could have been cleared by over-activity of the murine innate immune system (i.e., murine-derived monocytes and macrophages) in conjunction with more profound inflammatory response in the injured mice. Analysis of other tolerogenic immune cells, including myeloid derived suppressor cells from human and mouse, did not reveal significant changes in frequency (Supplementary Fig. S3, S4). Taken together, these data highlight the possibility that focused studies of suppressor cell functions could be designed in the humanized model but would need to be powered to account for high variability.

**Figure 6:**
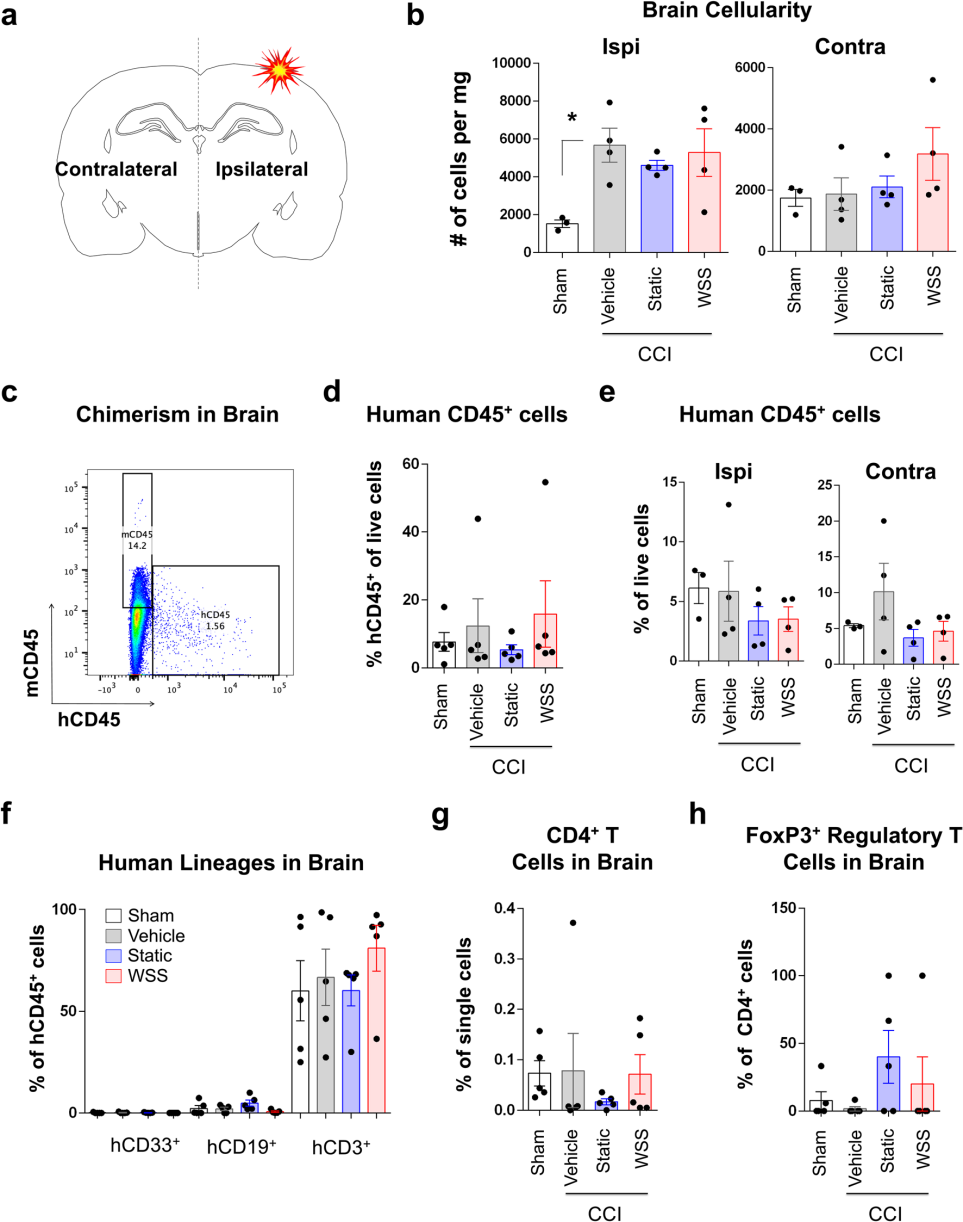
Human CD3^+^ T cells predominate in the brain. **(a)** Ipsilateral (injured) and contralateral (uninjured) hemispheres of the brain were processed for separate analysis. **(b)** Total cellularity of the ipsilateral hemisphere was increased by injury (One-way ANOVA with Holm-Sidak test, *p = 0.04). MSC therapy did not suppress expansion in cell numbers. Analysis of separate hemispheres included 3–4 mice in each group. **(c)** Human chimerism of dissociated brain tissue was assessed by flow cytometry. **(d, e)** Frequency of human CD45^+^ cells in total brain tissue (both hemispheres, n = 5 mice per group) and in separately processed hemispheres (n = 3–4 mice per group) is unaltered by injury. **(f)** Across all treatment groups, a small fraction of human myeloid or B cells were detected in the brain; whereas, T cells accounted for up to 100% of hCD45^+^ cells in several mice. **(g)** Injury appears to reduce CD4^+^ T cell frequency in the brain, though high variability exists between mice. **(h)** The fraction of human CD4^+^ T cells identifiable as FoxP3^+^ regulatory T cells is variable across treatment groups.

To define the effects of injury on the immune composition of the brain, the ipsilateral and contralateral hemispheres were processed for analyses of brain tissue (Fig. 6a). Total cellularity in the ipsilateral hemisphere was elevated by injury (Fig. 6b). Human hematopoietic cells populated the brain, and analysis of the whole brain and independently processed hemispheres revealed no significant difference in chimerism across treatment groups (Fig. 6c-e). T cells dominated the graft, accounting for up to 100% of the cells expressing hCD45, but did not appear to change in frequency in response to injury (Fig. 6f). Injury appeared to reduce CD4^+^ T cell frequency in the brain, though high variability existed between mice and no significant differences were found (Fig. 6g). The fraction of human CD4^+^ T cells identifiable as FoxP3^+^ regulatory T cells was also variable across treatment groups (Fig. 6h).

**Figure 7:**
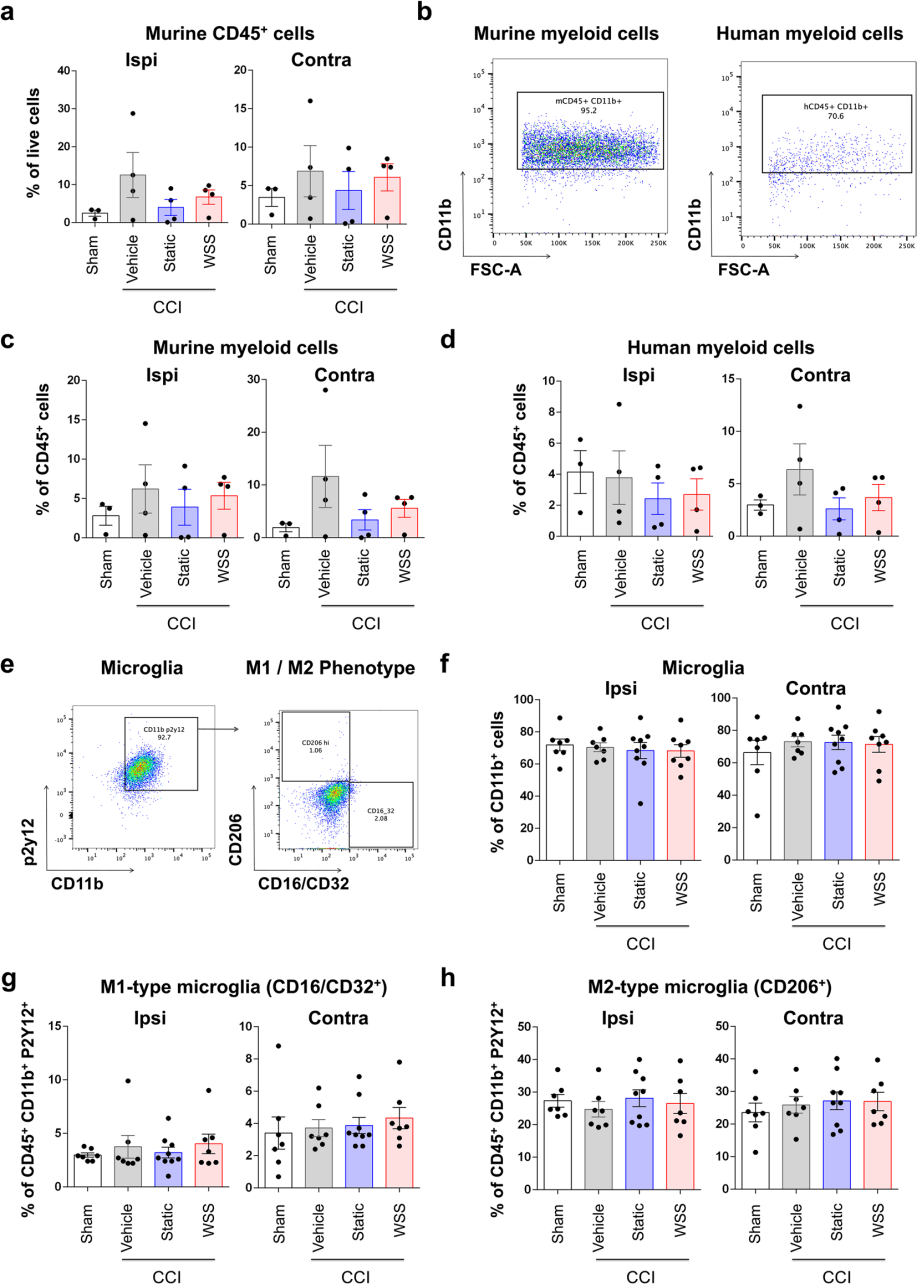
M1–M2-type microglial frequencies are not detectably altered 7 days after injury. **(a)** No significant change in murine CD45^+^ cells was observed in the brain. Hemispheres were evaluated separately in 3–4 mice per group. **(b)** Myeloid cells were detected by human and mouse reactive CD11b antibody. **(c, d)** Frequencies of mCD45^+^ CD11b^+^ and hCD45^+^ CD11b^+^ cells in the brain were not significantly different between treatment groups. **(e)** CD45^+^ CD11b^+^ P2Y12^+^ microglia were characterized as alternatively activated CD206^+^ M2-type microglia or inflammatory CD16/CD32^+^ M1-type microglia. **(f)** Frequencies of microglia in the ipsilateral and contralateral hemispheres appear unaffected after injury. Microglia were evaluated in 7–9 mice per group. **(g, h)** M1-type and M2-type microglia labeled by surface detection of CD16/CD32 and CD206, respectively, are not noticeably altered after injury.

Microglia have previously been shown to be activated at 7 days after CCI, as defined by immunohistological and morphometric analyses of brain in male and female mice^52^. To define the effects of injury in the humanized model, we examined reactivity and cell identity of microglia using several surface markers expressed on microglia, which we and others have used previously to segregate pro-inflammatory and anti-inflammatory phenotypes. CD11b^+^ myeloid cells were composed largely of murine host cells, and their frequencies were equivalent across treatment groups (Fig. 7a-d). Microglia expressing the p2y12 receptor comprised a subset of mCD45^+^ CD11b^+^ cells and were not found to be significantly different between treatment groups (Fig. 7e). Albeit binary and overly simplistic, additional markers of activation were used to assess subtype polarization toward the M2 phenotype (CD206) and M1 phenotype (Fc gamma receptor CD16/CD32). Using CD206 and CD16/CD32, we were unable to detect a change in frequencies of microglial subtypes in the injured brain (Fig. 7f–h). In our prior studies using similar flow cytometry phenotyping of resident and infiltrating immune cells in the mouse brain, we have found that the ratio of M1 to M2 microglia is elevated after injury and can be significantly reduced in injured mice receiving multipotent adult progenitor cells^53,54^. Key differences in approach could have contributed to disparate outcomes in these studies and the present data, including use of the microglia-specific p2y12 receptor here to exclude infiltrating monocytes and macrophages, as well as analysis of the brain only up to 5 days after injury in contrast to the 7 days used here. Alternatively, these data could suggest that the state of neuroinflammation in the humanized mice is altered relative to wild-type mice. We conclude that, at day 7 after injury, flow cytometry-based detection of CD206 and CD16/CD32 is insufficient to detect differences in microglia activation in the humanized mouse brain. This is consistent with an ongong conversation in the literature arguing that microglial activation is exquisitely dynamic and requires inclusion of a comprehensive panel of markers and/or gene expression to accurately assess polarization^11,55^.

## Discussion

The challenge of accurately modeling human immune response in animal models of neurotrauma has limited our ability to predict success of therapeutic interventions in clinical trials. Here, we report that a murine model harboring a humanized immune system can be used to examine the human immune response to brain injury in various organs of the body. Theoretically, the tractability of the model could enable assessment and comparison of therapies designed to minimize neuroinflammation and other sequelae of brain trauma. However, limitations include low-level human myeloid contribution to the periphery, variability in chimerism, and underlying GVHD. We also were unable to detect changes in microglia abundance and activation by flow cytometry alone. Nevertheless, we observed that TBI worsened GVHD-induced pathology in the bone marrow, which appeared to be partially alleviated by MSC therapy. No enhancement in MSC function by application of transient WSS pre-infusion could be detected. Our study represents a new perspective on how underlying immune reactivity such as exists in GVHD and autoimmune disorders could be exacerbated by injury.

Activity of the bone marrow has been shown to be altered by trauma in patients. Severe trauma induces endothelial hyperpermeability, hematopoietic stem and progenitor cell trafficking, anemia, inflammation, compensatory immunosuppression, and bone marrow dysfunction^56,57^. Specifically, Livingston and colleagues have described bone marrow failure, including reduced hematopoietic activity and severe growth defects in bone marrow stromal cells which are a critical component of the hematopoietic niche^5^. Given these alterations to the hematopoietic system, it is not surprising that post-injury infection is a leading cause of death in patients with TBI^9,58^. Having said this, our data in the C57BL/6 mouse does not reveal overt decimation of marrow, so it could be that histological evidence for these effects in human bone marrow biopsies would also appear relatively normal.

Although GVHD is better documented clinically in the skin, liver, and intestine, there is also evidence for GVHD in the bone marrow leading to cytopenias. TBI releases self-antigens into the peripheral circulation and lymph nodes via glymphatics and meningeal lymphatic vessels^59–61^. These self antigens stimulate naïve immune cells and trigger adaptive autoreactivity^62,63^. Autoreactive T-cells generated following TBI have the ability to co-activate B-cells, leading to autoantibody production in patients with TBI by seven days post-injury^64^. Because bone marrow stromal cells and other cells of the hematopoietic niche have poor engraftment potential, the osteoblasts and endothelium of the chimeric mouse are chiefly derived from the host^65^. Although these cell types are weak antigen presenters, they are believed to be targets of alloreactivity in the context of myeloablation and GVHD^66^. In addition, presentation of phagocytosed alloantigens by donor-derived antigen presenting cells by MHC Class II, and via cross presentation to MHC Class I, can support indirect niche destruction through alloreactive CD4^+^ T cells that secrete soluble factors. In the present study, the appearance of the bone marrow by gross observation and histological analysis strongly suggests that alloreactivity was a major contributor to marrow destruction and necrosis. The effect was more pronounced in injured mice that did not receive MSC therapy, suggesting that physiologic stress associated with injury could have exacerbated rejection of host marrow and/or niche components.

T cells are chief contributors to manifestation and severity of GVHD and act by secreting cytokines that lead to disease onset, especially in the context of cytokine storm^67,68^ such as that observed in TBI. Here, we show that bone marrow phenotype correlates tightly with T cell abundance. Mature CD4^+^ and CD8^+^ T lymphocytes undergo extensive migration from the peripheral blood to the bone marrow (and vice versa); thus, the bone marrow is a major reservoir for T cells^69^. Inflammatory syndromes characterized by uncontrolled activation and proliferation of T lymphocytes and macrophages often present early in life following viral infection or other immune activated conditions such as autoimmune disease and malignant lymphoma^70^. One such disease, hemophagocytic lymphohistiocytosis or macrophage activation syndrome, is caused by hypercytokinemia. Hemophagocytic lymphohistiocytosis has recently been modeled in humanized NSG mice and has shown that transplant regimes which enhance T cell development also lead to an abundance of hemosiderin-containing hemophagocytic histiocytes in the bone marrow, which is accompanied by lethal disease^71^. Consistent with this report, humanized NSG mice in our study also exhibited extensive histiocytosis in the marrow, and we found that mice with higher levels of human T cell contribution suffer a higher incidence of bone marrow loss. The identity of the cytokines driving marrow pathology is unclear, but putative candidates include those triggered by TBI such as the pro-inflammatory cytokines TNFα, IL-1β, IL-6, and IL-18 (inducer of IFN-γ), and some anti-inflammatory cytokines including IL-10^72^. Yet, some apparently pro-inflammatory Th1 cytokines can also have anti-inflammatory functions. For example, the Th1 secreted cytokine, IL-10, can modulate CD4^+^ T cell functions by down-regulation of IL-2^73^ but can also create a tolerogenic environment to allo-antigens^74^. Similarly, TNF-α priming can enhance Treg suppressive function to attenuate GVHD^75^. A future priority will be to elucidate how these cytokines contribute to recovery after TBI. Collectively, our data point to the bone marrow as an organ susceptible to inflammation and raises the possibility that elevated activation of T cells and associated inflammatory cytokine production could trigger autoreactivity of murine histiocytes toward niche cells of the marrow, further impairing the bone’s ability to support hematopoiesis.

CD4^+^ T cells, including Treg cells, were modestly decreased in the bone marrow and lymph nodes of injured vehicle control animals. The implications of this trend are somewhat difficult to predict in the absence of careful analysis of T cell phenotype and activity. Chronic and acute GVHD are characterized by activation and proliferation of conventional CD4^+^ T cells. Both natural and induced Treg cells restrain CD4^+^ conventional T cell proliferation and attenuate GVHD by multiple mechanisms, including IL-2, IL-10 and TGF-β^76^. Indeed, one of the most effective therapies for GVHD is to increase Treg frequency, such as by IL-2 therapy^77^. Thus, the decrease in Treg frequency observed in the injured vehicle control mice in our study suggests that TBI would produce greater inflammation in part by reducing Treg in the bone marrow and lymph nodes. However, a cautious approach at characterization of the Treg cells in the NSG mouse would necessarily include intracellular staining for IL-10 and in vitro measurement of Treg suppressor activity in T effector cell proliferation assays. Not surprisingly, MSCs have been shown to promote Treg expansion^78,79^ and substantial evidence supports their application to treat GVHD^80^. Of special relevance to the WSS-treated MSCs in our study is that MSC-derived TGF-β and prostaglandin E_2_ are key regulators of Treg cell induction^81,82^. Clinical consequences of cytokine perturbation in TBI are incompletely understood; however, based upon these data, it is feasible that settings of secondary inflammatory stimulus that upset marrow homeostasis like TBI could contribute to T-cell mediated hypercytokinemia resulting in loss of self-tolerance or immunodepression.

Several mechanisms could have contributed to improved bone marrow condition after MSC therapy. MSCs secrete a spectrum of soluble molecules that alter the local milieu to contribute to angiogenesis, cytoprotection, tissue repair, cell growth, and inflammatory suppression^83,84^. Likely through paracrine signaling, MSCs could modulate inflammatory T cell responses but could also suppress death of hematopoietic cells in the bone marrow. Consistent with the protective effects of MSCs on hematopoiesis, exogenous administration of secreted factors including platelet derived growth factor and TPO confer radioprotective effects on the bone marrow via reduction of apoptosis in multipotent hematopoietic stem and progenitor cells as well as mature blood lineages such as megakaryocytes^85^. Perhaps not coincidentally, TPO has also been shown to inhibit neuronal cell death^86^, further supporting existing evidence in the literature that paracrine signaling from MSCs produces positive pleiotropic effects on multiple organ systems.

Some limitations of the current mouse model could be viewed as unique opportunities to better understand the human immune system. For example, T cells dominated the graft, and many of these were CD4^+^ helper cells, which are found in greater numbers in the injured brain^14,15^. In humans, the frequencies of lymphoid and myeloid lineages vary across individuals, but typically the T lymphocyte population accounts for 10–25% of the leukocytes in the peripheral blood. We demonstrate a mean engraftment of approximately 50% T cells in the human graft for mice that met minimum requirements for chimerism. If only 50% of the white blood cells in the blood were human, that would translate to 25% human T cells in the humanized mouse blood. This T cell frequency falls within the range of a normal human peripheral blood sample. We suggest that the abundance of human T cells makes this a useful model for studying T cell contributions to recovery from neurotrauma. However, we acknowledge that the humanized model should be optimized and that certain limitations exist for study of certain immune cell subsets, such as myeloid lineages. Indeed, due to the rarity of human monocytes, modeling of the human innate immune response is likely not feasible. We were unable to resolve significant differences in neuroinflammation following injury, but ample evidence supports that immune response can play a role in recovery from traumatic CNS injury. In an independent report by Carpenter and colleagues, a similar humanization model showed that spinal cord injury resulted in different neuroinflammatory profiles before and after stable engraftment of the human immune system^87^. Neuroinflammation could be influenced by T cell activity, as it was shown in a non-humanized model that administration of T cells reactive to myelin protein antigens results in improved recovery in spinal cord and optic nerve injuries^88–90^. Stimulated T cells directly produce endogenous neurotrophins including BDNF^91^ and promote elevated neurotrophin production by neurons, outgrowth of neuronal axons, and modulation of microglia phenotype via secretion of IL-4, a prototypical Th2 cytokine^92–94^. Some of the protective function of T cells also derives from their effect on astrocytes. Study of two major cytokines produced by Th1 and Th2 cells, interferon-gamma and IL-4, respectively, suggests that a balanced Th1 and Th2 cytokine response is necessary to protect two chief functions of astrocytes, glutamate clearance and thiol secretion^92–95^. Together, numerous reports support a role for cytokines produced by Th1 and Th2 cells in neuroprotection and repair in CNS injury. Thus, we suggest that the robust engraftment of T cells in the humanized mouse presents a unique opportunity to identify the principal T cell subtypes that respond to TBI, elucidate how cells of the CNS engage with T cells following injury, and understand the role of cytokines in neural protection and repair.

Direct effects of marrow destruction on neurological outcomes of TBI are still unclear. Previous attempts to address the impact of the hematopoietic system on TBI outcomes have included approaches such as splenectomy. Splenectomy is a straightforward surgery that is tolerated well by rodents and has been shown to improve outcomes after TBI^96^. Destruction of bone marrow by irradiation or chemotherapy could be done prior to experimental TBI; however, mice are incredibly vulnerable to opportunistic infection, inflammatory response due to tissue damage, respiratory distress, dehydration, bleeding, and death in the weeks following myeloablation^97^. A more precise and informative strategy could include transplantation of T cell depleted hematopoietic progenitors or post-transplant depletion of immune cell subsets via antibody-based blocking, immunosuppressive agents, pretreatment with liposomal clodronate, and/or genetic targeting of the human immune cells^98,99^.

## Conclusion

Overall, the humanized mouse is a complex model system that is made more challenging to evaluate by variation in human chimerism and interactions between mouse and human cells. Nevertheless, with careful experimental design and cautious expectations, we believe that this tool could present unique opportunities for modeling human immune response to therapeutic interventions for neurological disease and injuries. We examined several immune cell subsets thought to be modulated in response to TBI and MSC therapy and have identified a compelling role for T cells in control of bone marrow fitness after neurotrauma. Our study supports premise for use of the humanized mouse model of TBI to identify components of the human immune system that drive positive response to cellular therapies, drug candidates, and methods for protection against nosocomial infection. More broadly, this research also argues for new appraisal of how autoimmunity and GVHD might be intensified by trauma.

## Supplementary information

Supplementary file1 (PDF 20193 kb)

## Data Availability

The data that support the findings of this study are available from the corresponding author upon reasonable request.

## References

[1] McDonald SJ, Sun M, Agoston DV, Shultz SR (2016). The effect of concomitant peripheral injury on traumatic brain injury pathobiology and outcome. J. Neuroinflamm..

[2] Xiong Y, Mahmood A, Chopp M (2013). Animal models of traumatic brain injury. Nat. Rev. Neurosci..

[3] Brooks GA, Martin NA (2014). Cerebral metabolism following traumatic brain injury: New discoveries with implications for treatment. Front. Neurosci..

[4] Fuchs A (2019). Trauma induces emergency hematopoiesis through IL-1/MyD88-dependent production of G-CSF. J. Immunol..

[5] Livingston DH (2003). Bone marrow failure following severe injury in humans. Ann. Surg..

[6] Nizamutdinov D, Shapiro LA (2017). Overview of traumatic brain injury: An immunological context. Brain Sci..

[7] Savitz SI, Cox CS (2016). Concise review: Cell therapies for stroke and traumatic brain injury: Targeting microglia. Stem cells.

[8] Xiao W (2011). A genomic storm in critically injured humans. J. Exp. Med..

[9] Hazeldine J, Lord JM, Belli A (2015). Traumatic brain injury and peripheral immune suppression: Primer and prospectus. Front. Neurol..

[10] Gentile LF (2014). A better understanding of why murine models of trauma do not recapitulate the human syndrome. Crit. Care Med..

[11] Jassam YN, Izzy S, Whalen M, McGavern DB, El Khoury J (2017). Neuroimmunology of traumatic brain injury: Time for a paradigm shift. Neuron.

[12] Kelso ML, Gendelman HE (2014). Bridge between neuroimmunity and traumatic brain injury. Curr. Pharm. Des..

[13] Liu Y-W, Li S, Dai S-S (2018). Neutrophils in traumatic brain injury (TBI): Friend or foe?. J. Neuroinflamm..

[14] Dressler J, Hanisch U, Kuhlisch E, Geiger KD (2007). Neuronal and glial apoptosis in human traumatic brain injury. Int. J. Legal Med..

[15] Holmin S, Söderlund J, Biberfeld P, Mathiesen T (1998). Intracerebral inflammation after human brain contusion. Neurosurgery.

[16] Baruch K, Schwartz M (2013). CNS-specific T cells shape brain function via the choroid plexus. Brain Behav. Immun..

[17] Cao C (2009). Aβ-specific Th2 cells provide cognitive and pathological benefits to Alzheimer's mice without infiltrating the CNS. Neurobiol. Dis..

[18] Nikolic WV (2007). Transcutaneous β-amyloid immunization reduces cerebral β-amyloid deposits without T cell infiltration and microhemorrhage. Proc. Natl. Acad. Sci..

[19] Fisher Y, Nemirovsky A, Baron R, Monsonego A (2010). T cells specifically targeted to amyloid plaques enhance plaque clearance in a mouse model of Alzheimer's disease. PLoS ONE.

[20] Prajeeth CK (2017). Effectors of Th1 and Th17 cells act on astrocytes and augment their neuroinflammatory properties. J. Neuroinflamm..

[21] Watanabe M (2016). Th1 cells downregulate connexin 43 gap junctions in astrocytes via microglial activation. Sci. Rep..

[22] Yang Y (2019). Acute traumatic brain injury induces CD4+ and CD8+ T cell functional impairment by upregulating the expression of PD-1 via the activated sympathetic nervous system. NeuroImmunoModulation.

[23] Baruch K (2016). PD-1 immune checkpoint blockade reduces pathology and improves memory in mouse models of Alzheimer's disease. Nat. Med..

[24] Helmy A, Carpenter KLH, Menon DK, Pickard JD, Hutchinson PJA (2010). The cytokine response to human traumatic brain injury: Temporal profiles and evidence for cerebral parenchymal production. J. Cereb. Blood Flow Metab..

[25] Roberts DJ (2013). Association between the cerebral inflammatory and matrix metalloproteinase responses after severe traumatic brain injury in humans. J. Neurotrauma.

[26] Cherry JD, Olschowka JA, O’Banion MK (2015). Arginase 1+ microglia reduce Aβ plaque deposition during IL-1β-dependent neuroinflammation. J. Neuroinflamm..

[27] Hinson HE, Rowell S, Schreiber M (2015). Clinical evidence of inflammation driving secondary brain injury: A systematic review. J. Trauma Acute Care Surg.

[28] Kawahara K (2012). Intracerebral microinjection of interleukin-4/interleukin-13 reduces β-amyloid accumulation in the ipsilateral side and improves cognitive deficits in young amyloid precursor protein 23 mice. Neuroscience.

[29] Shaftel SS (2007). Sustained hippocampal IL-1β overexpression mediates chronic neuroinflammation and ameliorates Alzheimer plaque pathology. J. Clin. Investig..

[30] Thelin EP (2018). Elucidating pro-inflammatory cytokine responses after traumatic brain injury in a human stem cell model. J. Neurotrauma.

[31] Zhao Y (2016). Wnt3a, a protein secreted by mesenchymal stem cells is neuroprotective and promotes neurocognitive recovery following traumatic brain injury. Stem cells.

[32] Peng W (2015). Systematic review and meta-analysis of efficacy of mesenchymal stem cells on locomotor recovery in animal models of traumatic brain injury. Stem Cell Res Ther.

[33] Diaz MF (2017). Biomechanical forces promote immune regulatory function of bone marrow mesenchymal stromal cells. Stem Cells.

[34] Lee HJ (2017). Focal adhesion kinase signaling regulates anti-inflammatory function of bone marrow mesenchymal stromal cells induced by biomechanical force. Cell. Signal..

[35] Diaz MF, Evans SM, Olson SD, Cox CS, Wenzel PL (2017). A co-culture assay to determine efficacy of TNF-α suppression by biomechanically induced human bone marrow mesenchymal stem cells. Bio-Protocol.

[36] Kota DJ (2017). Prostaglandin E2 indicates therapeutic efficacy of mesenchymal stem cells in experimental traumatic brain injury. Stem cells.

[37] Li N, Diaz MF, Wenzel PL (2015). Application of fluid mechanical force to embryonic sources of hemogenic endothelium and hematopoietic stem cells. Methods Mol. Biol..

[38] 38Dominici, M. *et al.* Minimal criteria for defining multipotent mesenchymal stromal cells. The International Society for Cellular Therapy position statement. *Cytotherapy* **8**, 315–317 (2006). 10.1080/14653240600855905 16923606

[39] Lu H (2004). Microfluidic shear devices for quantitative analysis of cell adhesion. Anal. Chem..

[40] Diaz MF (2015). Biomechanical forces promote blood development through prostaglandin E2 and the cAMP-PKA signaling axis. J. Exp. Med..

[41] Shultz LD (2005). Human lymphoid and myeloid cell development in NOD/LtSz-scid IL2Rγnull mice engrafted with mobilized human hemopoietic stem cells. J. Immunol..

[42] Choi B (2011). Human T cell development in the liver of humanized NOD/SCID/IL-2Rγnull(NSG) mice generated by intrahepatic injection of CD34+ human (h) cord blood (CB) cells. Clin Immunol.

[43] Ishikawa F (2005). Development of functional human blood and immune systems in NOD/SCID/IL2 receptor γ chain null mice. Blood.

[44] Cowen EW (2016). Graft-vs-host disease JAMA dermatology patient page. JAMA Dermatol.

[45] Geraghty NJ (2019). Increased splenic human CD4+:CD8+ T cell ratios, serum human interferon-γ and intestinal human interleukin-17 are associated with clinical graft-versus-host disease in humanized mice. Transpl. Immunol..

[46] Bagnara D (2011). A novel adoptive transfer model of chronic lymphocytic leukemia suggests a key role for T lymphocytes in the disease. Blood.

[47] Ehx G (2018). Xenogeneic graft-versus-host disease in humanized NSG and NSG-HLA-A2/HHD mice. Front. Immunol..

[48] Li M (2011). Immediate splenectomy decreases mortality and improves cognitive function of rats after severe traumatic brain injury. J. Trauma Acute Care Surg..

[49] Seifert HA (2012). A transient decrease in spleen size following stroke corresponds to splenocyte release into systemic circulation. J. Neuroimmune Pharmacol..

[50] Nishi Y (2019). Adipose tissue-derived mesenchymal stem cells ameliorate bone marrow aplasia related with graft-versus-host disease in experimental murine models. Transpl. Immunol..

[51] Søndergaard H, Kvist PH, Haase C (2013). Human T cells depend on functional calcineurin, tumour necrosis factor-α and CD80/CD86 for expansion and activation in mice. Clin. Exp. Immunol..

[52] Villapol S, Loane DJ, Burns MP (2017). Sexual dimorphism in the inflammatory response to traumatic brain injury. Glia.

[53] Bedi SS, Smith P, Hetz RA, Xue H, Cox CS (2013). Immunomagnetic enrichment and flow cytometric characterization of mouse microglia. J. Neurosci. Methods.

[54] Walker PA (2012). Intravenous multipotent adult progenitor cell therapy after traumatic brain injury: Modulation of the resident microglia population. J. Neuroinflamm..

[55] Ransohoff RM (2016). A polarizing question: Do M1 and M2 microglia exist?. Nat. Neurosci..

[56] Daglas M, Adlard PA (2018). The involvement of iron in traumatic brain injury and neurodegenerative disease. Front. Neurosci..

[57] Kumar M, Bhoi S (2016). Impaired hematopoietic progenitor cells in trauma hemorrhagic shock. J. Clin. Orthopaed. Trauma.

[58] Lepelletier, D. *et al.* Retrospective analysis of the risk factors and pathogens associated with early-onset ventilator-associated pneumonia in surgical-ICU head-trauma Patients. *J. Neurosurg. Anesthesiol.* **22** (2010). 10.1097/ANA.0b013e3181bdf52f 20027012

[59] Absinta, M. *et al.* Human and nonhuman primate meninges harbor lymphatic vessels that can be visualized noninvasively by MRI. *eLife* **6**, e29738 (2017). 10.7554/eLife.29738 PMC5626482 28971799

[60] Mondello, S. *et al.* Blood-based protein biomarkers for the management of traumatic brain injuries in adults presenting to emergency departments with mild brain injury: A living systematic review and meta-analysis. *J. Neurotrauma* (2017). 10.1089/neu.2017.5182 PMC8054517 29020853

[61] Plog BA (2015). Biomarkers of traumatic injury are transported from brain to blood via the glymphatic system. J. Neurosci..

[62] Cox AL (2006). An investigation of auto-reactivity after head injury. J. Neuroimmunol..

[63] Harling-Berg C, Knopf PM, Merriam J, Cserr HF (1989). Role of cervical lymph nodes in the systemic humoral immune response to human serum albumin microinfused into rat cerebrospinal fluid. J. Neuroimmunol..

[64] Chenouard A (2015). Phenotype and functions of B cells in patients with acute brain injuries. Mol. Immunol..

[65] Awaya N, Rupert K, Bryant E, Torok-Storb B (2002). Failure of adult marrow-derived stem cells to generate marrow stroma after successful hematopoietic stem cell transplantation. Exp. Hematol..

[66] Szyska M, Na I-K (2016). Bone marrow GvHD after allogeneic hematopoietic stem cell transplantation. Front. Immunol..

[67] Mohty M (2005). Inflammatory cytokines and acute graft-versus-host disease after reduced-intensity conditioning allogeneic stem cell transplantation. Blood.

[68] Piper C, Drobyski WR (2019). Inflammatory cytokine networks in gastrointestinal tract graft vs host disease. Front Immunol.

[69] Di Rosa F, Pabst R (2005). The bone marrow: A nest for migratory memory T cells. Trends Immunol..

[70] Jordan, M. B. *et al.* Challenges in the diagnosis of hemophagocytic lymphohistiocytosis: Recommendations from the North American Consortium for Histiocytosis (NACHO). *Pediatric Blood Cancer* e27929 (2019). 10.1002/pbc.27929 PMC7340087 31339233

[71] Yoshihara S (2019). Posttransplant hemophagocytic lymphohistiocytosis driven by myeloid cytokines and vicious cycles of T-cell and macrophage activation in humanized mice. Front. Immunol..

[72] Helmy A, De Simoni M-G, Guilfoyle MR, Carpenter KLH, Hutchinson PJ (2011). Cytokines and innate inflammation in the pathogenesis of human traumatic brain injury. Prog. Neurobiol..

[73] Taga K, Mostowski H, Tosato G (1993). Human interleukin-10 can directly inhibit T-cell growth. Blood.

[74] Groux H, Bigler M, de Vries JE, Roncarolo MG (1996). Interleukin-10 induces a long-term antigen-specific anergic state in human CD4+ T cells. J. Exp. Med..

[75] Krenger W, Ferrara JLM (1996). Graft-versus-host disease and the Th1/Th2 paradigm. Immunol. Res..

[76] Shin H-J (2011). Rapamycin and IL-2 reduce lethal acute graft-versus-host disease associated with increased expansion of donor type CD4+CD25+Foxp3+ regulatory T cells. Blood.

[77] Matsuoka, K.-I. *et al.* Low-dose interleukin-2 therapy restores regulatory T cell homeostasis in patients with chronic graft-versus-host disease. *Sci. Transl. Med.* **5**, 179ra143 (2013). 10.1126/scitranslmed.3005265 PMC3686517 23552371

[78] Aggarwal S, Pittenger MF (2005). Human mesenchymal stem cells modulate allogeneic immune cell responses. Blood.

[79] Di Ianni M (2008). Mesenchymal cells recruit and regulate T regulatory cells. Exp. Hematol..

[80] Shi M, Liu ZW, Wang FS (2011). Immunomodulatory properties and therapeutic application of mesenchymal stem cells. Clin. Exp. Immunol..

[81] English K (2009). Cell contact, prostaglandin E2 and transforming growth factor beta 1 play non-redundant roles in human mesenchymal stem cell induction of CD4+CD25 highforkhead box P3+ regulatory T cells. Clin. Exp. Immunol..

[82] Prevosto C, Zancolli M, Canevali P, Zocchi MR, Poggi A (2007). Generation of CD4+ or CD8+ regulatory T cells upon mesenchymal stem cell-lymphocyte interaction. Haematologica.

[83] Bassi ÊJ, de Almeida DC, Moraes-Vieira PM, Câmara NO (2012). Exploring the role of soluble factors associated with immune regulatory properties of mesenchymal stem cells. Stem Cell Rev.

[84] Ma S, Xie N, Li W, Yuan B, Shi Y, Wang Y (2014). Immunobiology of mesenchymal stem cells. Cell Death Differ.

[85] Ye JY (2010). Platelet-derived growth factor enhances platelet recovery in a murine model of radiation-induced thrombocytopenia and reduces apoptosis in megakaryocytes via its receptors and the PI3-k/Akt pathway. Haematologica.

[86] Li L (2020). c-Mpl and TPO expression in the human central nervous system neurons inhibits neuronal apoptosis. Aging.

[87] Carpenter RS (2019). Human immune cells infiltrate the spinal cord and impair recovery after spinal cord injury in humanized mice. Sci. Rep..

[88] Hauben E (2001). Posttraumatic therapeutic vaccination with modified myelin self-antigen prevents complete paralysis while avoiding autoimmune disease. J. Clin. Investig..

[89] Hauben E (2000). Autoimmune T cells as potential neuroprotective therapy for spinal cord injury. Lancet.

[90] Moalem G (1999). Autoimmune T cells protect neurons from secondary degeneration after central nervous system axotomy. Nat. Med..

[91] Kerschensteiner M (1999). Activated human T cells, B cells, and monocytes produce brain-derived neurotrophic factor in vitro and in inflammatory brain lesions: A neuroprotective role of inflammation?. J. Exp. Med..

[92] Fenn AM, Hall JCE, Gensel JC, Popovich PG, Godbout JP (2014). IL-4 signaling drives a unique arginase+/IL-1β+ microglia phenotype and recruits macrophages to the inflammatory CNS: Consequences of age-related deficits in IL-4Rα after traumatic spinal cord injury. J. Neurosci..

[93] Walsh JT (2015). MHCII-independent CD4+ T cells protect injured CNS neurons via IL-4. J. Clin. Investig..

[94] Zhao X (2015). Neuronal interleukin-4 as a modulator of microglial pathways and ischemic brain damage. J. Neurosci..

[95] Garg SK, Kipnis J, Banerjee R (2009). IFN-γ and IL-4 differentially shape metabolic responses and neuroprotective phenotype of astrocytes. J. Neurochem..

[96] Li, M. *et al.* Immediate splenectomy decreases mortality and improves cognitive function of rats after severe traumatic brain injury. *J. Trauma Acute Care Surg.* **71** (2011). 10.1097/TA.0b013e3181f30fc9 21248654

[97] Duran-Struuck R, Dysko RC (2009). Principles of bone marrow transplantation (BMT): Providing optimal veterinary and husbandry care to irradiated mice in BMT studies. J. Am. Assoc. Lab. Anim. Sci..

[98] Aversa F, Pierini A, Ruggeri L, Martelli MF, Velardi A (2019). The evolution of T cell depleted haploidentical transplantation. Front Immunol.

[99] van Rooijen, N. & Hendrikx, E. In *Liposomes: Methods and Protocols, Volume 1: Pharmaceutical Nanocarriers* (ed. Weissig, V.) 189–203 (Humana Press, 2010).

