# Supplementary material for: Injury intensifies T cell mediated graft-versus-host disease in a humanized model of traumatic brain injury
Source: Sci Rep. 2020 Jul 1;10:10729. doi: 10.1038/s41598-020-67723-x (PMC7330041; doi:10.1038/s41598-020-67723-x)
Supplement: Supplementary file 1 — Supplementary file1 (PDF 20193 kb) [file 41598_2020_67723_MOESM1_ESM.pdf]

Injury intensifies T cell mediated graft-versus-host disease in a humanized model of traumatic brain injury

Miguel F. Diaz[1,2,3], Paulina D. Horton[1,2,3], Akshita Kumar[1], Megan Livingston[1,2,3], Amina Mohammadalipour[3], Hasen Xue[1], Max A. Skibber[1,4], Adesuwa Ewere[1,2, #], Naama E. Toledano Furman[1], Kevin R. Aroom[1], Songlin Zhang[5], Brijesh S. Gill[4], Charles S. Cox, Jr.[1,2], and Pamela L. Wenzel[1,2,3,*]

Supplementary Table S1. Antibody panels used for analysis of chimerism and immune cell identification.

Human-mouse chimerism, plus human lineages
anti-human CD45-APC-Cy7 (2D1)
anti-mouse CD45.1-FITC (A20)
anti-human CD19-PerCP-Cy5.5 (HIB19)
anti-human CD3-APC (UCHT1)
anti-human CD33-PE (WM53)
DAPI

Human T regulatory cells (True-Nuclear Human Treg Flow Kit)
anti-human FOXP3-Alexa 488 (150D)
anti-human CD4 PE-Cy5
anti-human CD25-PE

Murine MDSCs
anti-mouse Ly-6C-APC-Cy7 (AL-21)
anti-mouse Ly-6G-PE (1A8)
anti-mouse/human CD11b PerCP-Cy5.5 (M1/70)
anti-mouse Gr1/Ly-6G-APC (RB6-8C5)
anti-mouse CD45.1-FITC (A20)
DAPI

Human MDSCs
anti-human CD14-APC-Cy7 (MΦP9)
anti-human CD15-PE (HI98)
anti-human CD11b/MAC-1-FITC (ICRF44)
anti-human HLA-DR-APC (G46-6)
DAPI

Murine microglia in brain
anti-mouse/human CD11b-AF488 (M1/70)
anti-human CD45-PE (2D1)
anti-mouse CD16/32-PerCP-Cy5.5 (93)
anti-mouse CD206-APC (C068C2)
anti-mouse CD45-APC-Cy7 (30-F11)
anti-human/rat/mouse P2RY12 polyclonal (#APR-020)
donkey anti-rabbit IgG-BV421
Ghost dye-BV510

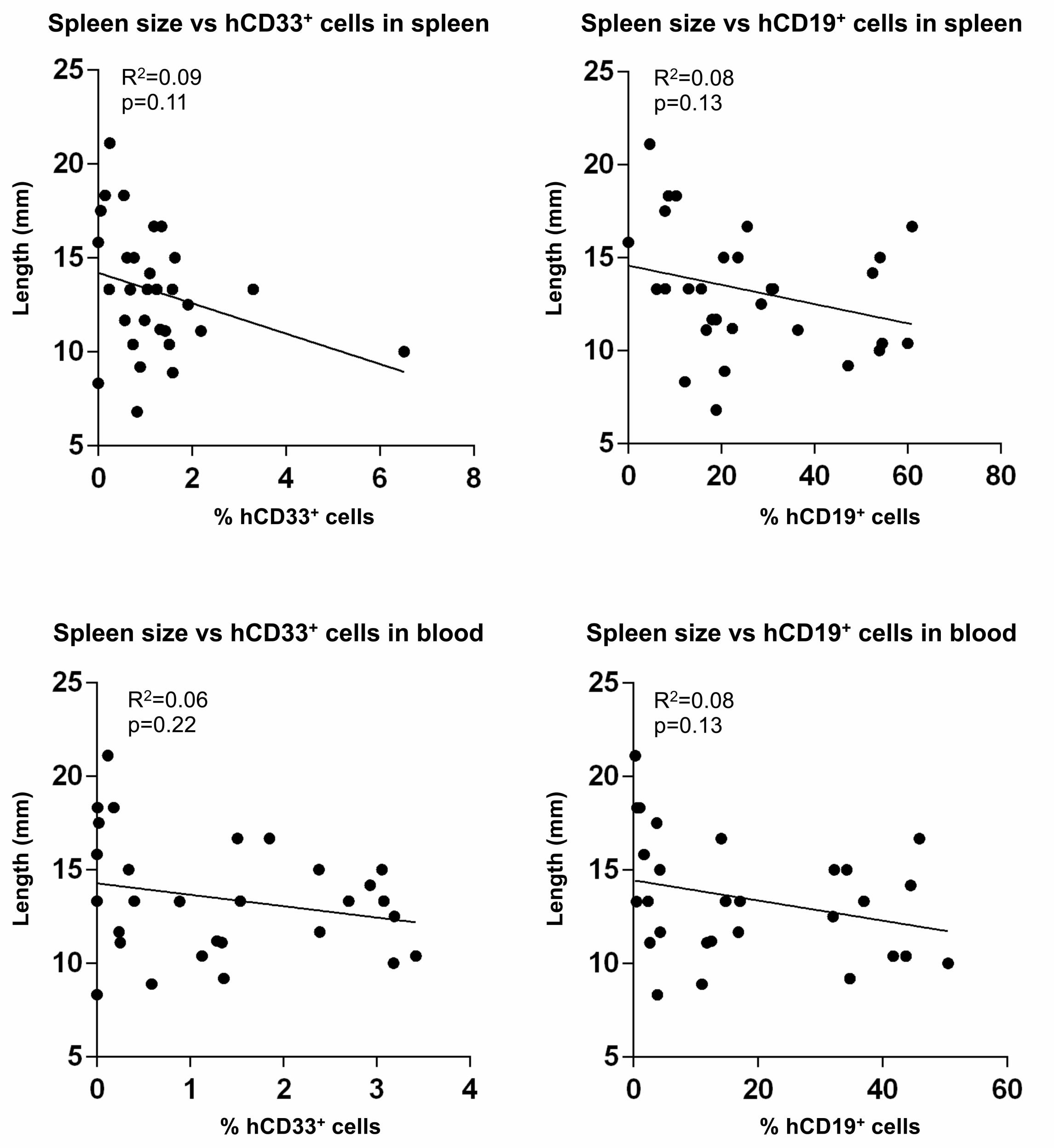

Supplementary Figure S1. Spleen size relative to human myeloid and B cell chimerism in spleen and peripheral blood. No significant relationship exists between length of the spleen and $hCD33^+$ myeloid or $hCD19^+$ B cells (n=29 mice).

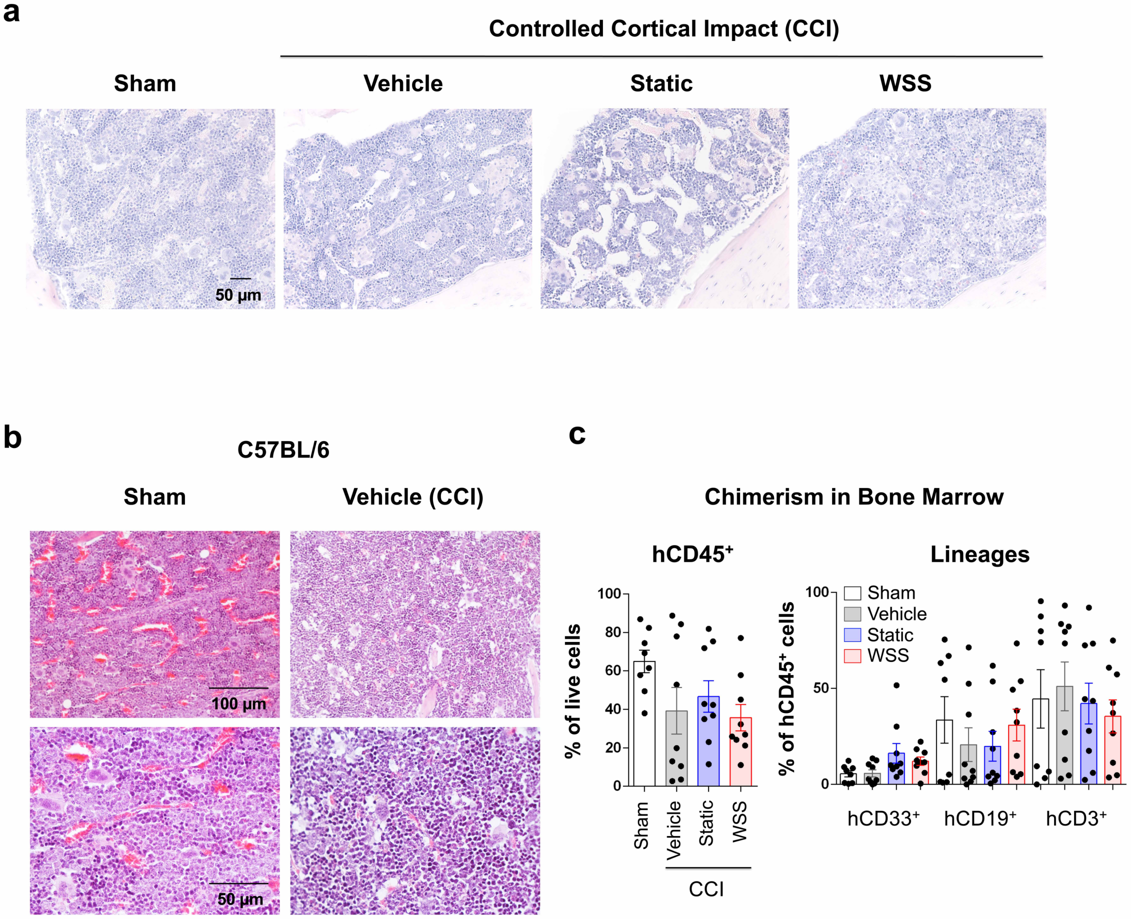

Supplementary Figure S2. Regional loss of red marrow in the long bones is restricted to humanized NSG mice. (a) Tibia from each treatment group stained by hematoxylin and eosin were similar in composition to femurs. **(b)** C57BL/6 bone marrow did not exhibit the same defects detected in marrow of transplanted NSG mice. **(c)** Frequencies of $hCD45^+$ and $lineage^+$ cells in the marrow were not significantly impacted (n=8-9 mice per group).

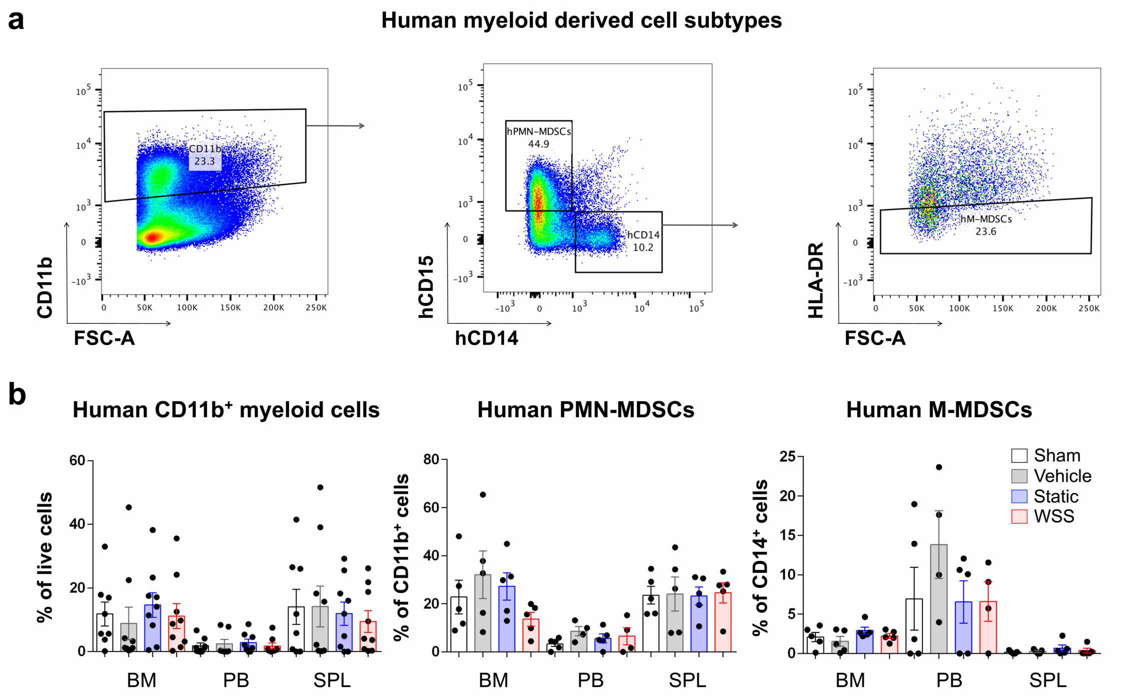

Supplementary Figure S3. Human myeloid derived suppressor cell subtypes are not significantly altered by injury. (a) Human CD11b^{+} myeloid cells were categorized as polymorphonuclear (PMN) by positivity for hCD15 or as monocytic (M) if hCD14^{+} HLA-DR^{-}. **(b)** Human myeloid suppressor cell frequencies were not significantly altered 7 days after injury. CD11b+ cell frequencies were determined in 8-9 mice per group in the bone marrow, peripheral blood, and spleen; whereas, MDSC frequencies were measured in 5 mice per group.

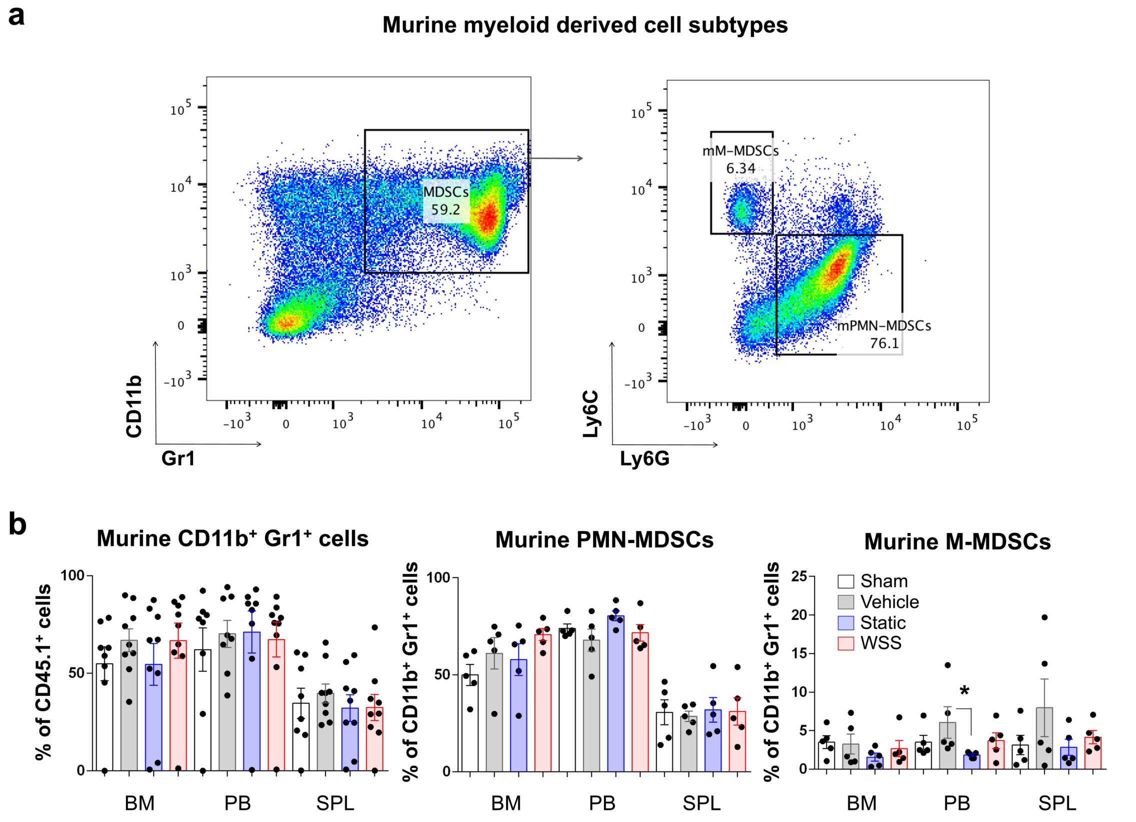

Supplementary Figure S4. Frequencies of murine myeloid derived cells are relatively unchanged 7 days after neurotrauma. (a) CD11b^{+} Gr1^{+} myeloid cells were identified by Ly6G positivity as polymorphonuclear (PMN) or as monocytic (M) by Ly6C positivity. **(b)** Frequencies of MDSCs and PMN-MDSCs were modestly increased in the bone marrow. CD11b+ Gr1+ cells were determined in 8-9 mice per group in the bone marrow, peripheral blood, and spleen; whereas, MDSC frequencies were measured in 5 mice per group.
